# Traffic of Secondary Metabolites to Cell Surface in the Red Alga *Laurencia dendroidea* Depends on a Two-Step Transport by the Cytoskeleton

**DOI:** 10.1371/journal.pone.0063929

**Published:** 2013-05-21

**Authors:** Vanessa M. Reis, Louisi S. Oliveira, Raoni M. F. Passos, Nathan B. Viana, Cláudia Mermelstein, Celso Sant'Anna, Renato C. Pereira, Wladimir C. Paradas, Fabiano L. Thompson, Gilberto M. Amado-Filho, Leonardo T. Salgado

**Affiliations:** 1 Diretoria de Pesquisas, Instituto de Pesquisas Jardim Botânico do Rio de Janeiro, Rio de Janeiro, RJ, Brazil; 2 Instituto de Biologia, Universidade Federal do Rio de Janeiro, Rio de Janeiro, RJ, Brazil; 3 Instituto de Física, Universidade Federal do Rio de Janeiro, Rio de Janeiro, RJ, Brazil; 4 Instituto de Ciências Biomédicas, Universidade Federal do Rio de Janeiro, Rio de Janeiro, RJ, Brazil; 5 Instituto Nacional de Metrologia, Qualidade e Tecnologia – Inmetro, Diretoria de Programas – Dipro, Duque de Caxias, RJ, Brazil; 6 Departamento de Biologia Marinha, Universidade Federal Fluminense, Niterói, RJ, Brazil; Semmelweis University, Hungary

## Abstract

In *Laurencia dendroidea,* halogenated secondary metabolites are primarily located in the vacuole named the *corps en cerise* (*CC*). For chemical defence at the surface level, these metabolites are intracellularly mobilised through vesicle transport from the *CC* to the cell periphery for posterior exocytosis of these chemicals. The cell structures involved in this specific vesicle traffic as well as the cellular structures related to the positioning and anchoring of the *CC* within the cell are not well known. Here, we aimed to investigate the role of cytoskeletal elements in both processes. Cellular and molecular assays were conducted to i) determine the ultrastructural apparatus involved in the vesicle traffic, ii) localise cytoskeletal filaments, iii) evaluate the role of different cytoskeletal filaments in the vesicle transport, iv) identify the cytoskeletal filaments responsible for the positioning and anchoring of the *CC*, and v) identify the transcripts related to cytoskeletal activity and vesicle transport. Our results show that microfilaments are found within the connections linking the *CC* to the cell periphery, playing an essential role in the vesicle traffic at these connections, which means a first step of the secondary metabolites transport to the cell surface. After that, the microtubules work in the positioning of the vesicles along the cell periphery towards specific regions where exocytosis takes place, which corresponds to the second step of the secondary metabolites transport to the cell surface. In addition, microtubules are involved in anchoring and positioning the *CC* to the cell periphery. Transcriptomic analysis revealed the expression of genes coding for actin filaments, microtubules, motor proteins and cytoskeletal accessory proteins. Genes related to vesicle traffic, exocytosis and membrane recycling were also identified. Our findings show, for the first time, that actin microfilaments and microtubules play an underlying cellular role in the chemical defence of red algae.

## Introduction

Marine seaweeds produce a diverse array of secondary metabolites, including terpenes, sterols, polyphenols, acetogenins and others [1]. Recent studies have revealed that some of these chemicals may act as chemical defences able to deter a broad range of natural enemies, including competitors [2], epiphytes [3], herbivores [4], and others [5]. Among the red seaweeds, the genus *Laurencia* produces the richest variety of secondary metabolites, generating more than 500 previously described compounds [1], [6]. For example, Brazilian *Laurencia dendroidea*, previously named *Laurencia obtusa* [7], is known for the biosynthesis of many halogenated compounds, such as the sesquiterpene elatol, that exhibit several ecological roles such as strong anti-fouling [8], [9] and anti-herbivory activities [10].

In general, the phenotypic expression or ecological roles of the seaweed secondary metabolites are well known, and they have been described in recent important reviews [11]–[13]. In contrast, the cellular processes involved in the exudation of macroalgal secondary metabolites and their further action in chemical defence are poorly understood, and there is a lack of information linking their chemistry, genetics and structural biology. However, studies of the production, transport, and deposition of secondary metabolites provide another relevant level of resolution and need to be considered in the evaluation of these chemicals and their patterns of distribution [14], [15].

Most of the published data are related to the localisation of secondary metabolite storage sites, such as gland cells and *corps en cerise* vacuoles (*CC*) [16]–[19], but the discovery of cellular processes related to chemical defence may provide a greater understanding of how secondary metabolites are used in the survival strategies of algae. For instance, an alga that can defend itself against epiphytic pathogens and fouling organisms may have specific ways to transport certain stored secondary metabolites to the cell wall surface [19]–[22]. Recently, it was shown that the red macroalga *Callophycus serratus* increases the concentrations of secondary metabolites on surface areas to which the fungus *Lindra thalassiae* is adhered, exhibiting a specific chemical defence response to a pathogenic organism [23]. In *L. dendroidea*, the transfer of secondary metabolites from storage organelles (*CC*) to the cell surface occurs through vesicle transport and subsequent exocytosis [19], [20]. The importance of such vesicle transport to protection against bacterial fouling has been confirmed, as demonstrated by the induction of vesicle traffic in response to epiphytic bacteria [21].

Nevertheless, little is known concerning the cellular machinery involved in the vesicle traffic of *L. dendroidea* or any other red macroalga. It has been shown that vesicle transport occurs along membranous tubular structures connecting the *CC* organelle to the cell periphery [24]. However, little evidence describing the involvement of cytoskeletal elements in this process has been found [19]. Based on the analysis of time-lapse microscopy images and the speed of vesicle transport (∼40 nm.s^−1^), it was suggested that the connections between the *CC* and the cell periphery are formed internally by cytoskeletal filaments, possibly actin filaments [19]. To the best of our knowledge, there is only one study regarding the relationship between the cytoskeleton and secondary metabolism in algae, in the brown alga *Hormosira banksii* [24]. In this alga, both actin microfilaments and microtubules are necessary to move the physodes, a phenolic storage organelle [24]. For some red algae species, both microtubules and actin filaments are involved in the movement of organelles and vesicles [25], [26]. However, there is still no report addressing the role of the cytoskeleton in the relationship between secondary metabolism and the dynamic process of chemical defence in macroalgae.

Therefore, our aim was to study the involvement of the cytoskeleton in the dynamic intra-cellular traffic of halogenated compounds, relating it to the process of chemical defence in *L. dendroidea.* In addition, we investigated the participation of the cytoskeleton in the anchorage of the unusual storage vacuole *CC*. We performed *in vitro* assays using cytoskeleton stabilising and destabilising drugs, fluorescent actin labelling, ultrastructural analysis by scanning electron microscopy and microscopy of organelle manipulation with optical tweezers. At the molecular level, a transcriptomic analysis was conducted to characterise the genes related to the specific machinery involved in the secretory pathway and cytoskeleton dynamics in *L. dendroidea*.

## Materials and Methods

### Algae Collection and Cultivation

Samples of *Laurencia dendroidea* were collected on rocky shores in the mid-littoral zone at Praia Rasa (Armação dos Búzios, Rio de Janeiro, Brazil, 22°43′58″S, 41°57′25″W). No specific permissions were required for collection of *L. dendroidea* specimens. The location is not privately-owned or protected in any way and the field studies did not involve endangered or protected species. In the laboratory, fresh individuals of this macroalga were cleaned of epiphytes to produce clones that were cultivated in Von Stosch’s enriched seawater [27] supplemented with germanium dioxide at 1.9 mM [28]. Physical conditions were maintained as follows: a temperature of approximately 20°C, a light:dark cycle of 14 h:10 h and constant light intensity (60 µmol.m^−2^.s^−1^). Unialgal cultivation was performed according to Oliveira et al. [29].

### Actin Labelling

At this assay, some clones of algae were directly subjected to the actin labelling using phalloidin-FITC, while other clones were pre-treated with latrunculin B before the actin labelling to determine the effect of the drug on the actin cytoskeleton and also to confirm the specificity of phalloidin-FITC. The individuals of *L. dendroidea* were kept in cultivation with latrunculin B (Lat) (Sigma Aldrich) at 1.0 µM for 3 days. For the labelling procedure, the samples of *L. dendroidea* were fixed with 0.66 M formaldehyde and 10 mM glutaraldehyde diluted in sea water using a microwave oven for 4 seconds and then immersed in ice. The samples were then treated for 40 minutes with 1% cellulase diluted in MES buffer (pH 5.0) and protease inhibitors. After enzymatic digestion, the material was washed and treated with 8 mM Triton X100 for 40 minutes for cell membrane permeabilisation. Samples were washed and incubated with phalloidin-FITC (Sigma Aldrich, diluted 1∶100 in PBS) for 24 h. The algae fragments were visualised with an Olympus BX51 fluorescence optical microscope using a 488 nm excitation wavelength. Digitised images were obtained with a CoolSnap-Pro Color RS Photometrics CCD camera. Images obtained by using conventional fluorescence microscopy were processed with ImageJ software [30] (details in “Digital processing of optical microscopy images”). Algae fragments were also observed with a laser scanning confocal microscope (LSCM) Leica SPE, where two excitation wavelengths were used, 405 nm and 488 nm. The analysis with 405 nm laser wavelength was performed to shown chloroplasts and CC auto-fluorescence, with emission ranging from 650 to 750 nm. The analysis with 488 nm laser wavelength was performed to visualize the phalloidin-FITC fluorescence, with emission ranging from 500 to 550 nm.

### Destabilisation of Actin Microfilaments

Clones of *L. dendroidea* were kept in cultivation with latrunculin B (Lat) (Sigma Aldrich) at 0.1 and 1.0 µM for 3 days. Samples were analysed with an Olympus BX51 fluorescence optical microscope (100X 1.3 N.A. objective lens) to assess possible differences in vesicle transport and in the structure of membranous tubular connections linking the *CC* to the cell periphery. Samples were also analysed by using optical tweezers during microscopy (see methodology below). Digitised images were obtained with a CoolSnap-Pro Color RS Photometrics CCD camera and processed with ImageJ software (details in “Digital processing of optical microscopy images”).

### Depolymerisation and Stabilisation of Microtubules

A depolymerising drug (colchicine, Sigma Aldrich) was used to evaluate the role of microtubules in vesicle transport and in the structure of the tubular membranous connections. Fragments of *L. dendroidea* were incubated with colchicine at 0.15 and 1.5 mM for four hours. Samples were analysed with an Olympus BX51 optical microscope (100X 1.3 N.A. objective lens). Digitised images were obtained with a CoolSnap-Pro Color RS Photometrics CCD camera and were processed with ImageJ software (details in “Digital processing of optical microscopy images”). Samples were also analysed by microscopy using optical tweezers (see methodology below).

A microtubule stabilising drug (paclitaxel, Sigma Aldrich) was used to evaluate the role of microtubules in vesicle transport and in the structure of the tubular membranous connections. Fragments of *L. dendroidea* were incubated in culture medium mixed with paclitaxel at 0.012 mM and 0.12 mM for 3 hours. The algal tissue was processed for scanning electron microscopy (see method description below) to analyse the cytoskeletal network after paclitaxel treatment.

### Simultaneous Destabilisation of Actin Filaments and Depolimerization of Microtubules

Concerning the determination of the origin site of the vesicles transported through the tubular membranous connections, an experiment affecting both cytoskeleton filaments was performed, where the algae was simultaneously incubated with the drugs Lat (at 1 µM) and colchicine (at 1.5 mM). The changes in vesicle traffic were registered with an Olympus BX51 optical microscope (100X 1.3 N.A. objective lens). Digitised images were obtained with a CoolSnap-Pro Color RS Photometrics CCD camera and were processed with ImageJ software (details in “Digital processing of optical microscopy images”).

### Scanning Electron Microscopy

Algae samples were fixed in 10 mM glutaraldehyde (in 100 mM sodium cacodylate buffer, pH 7.4) and post-fixed with 40 mM osmium tetroxide for 1 h at room temperature. They were then dehydrated in an ethanol series (35%, 70%, 80%, 90%, and 100%). Samples were dried using a critical point dryer (Bal-Tec SPD 030), fractured using tape to peel off the superficial wall layers of the cells, mounted in aluminium stubs, and coated with a thin gold layer (10 nm) using a sputter coater (Bal-Tec SCD 050). Images were obtained with a Zeiss EVO 40 SEM operated at an accelerating voltage of 10 kV.

### Optical Tweezers Microscopy

Optical tweezers microscopy was used to manipulate the *CC* in living cells to determine the strength of the attachment of the *CC* to the cell periphery. The results obtained were related to the specific drug treatments (colchicine and latrunculin) used to change the cytoskeletal polymerisation properties.

The optical trap (OT) was constructed by illuminating a Nikon Plan Apo NA 1.4 objective placed in a Nikon Eclipse TE-300 microscope (Nikon, Melville, NY) with an Nd-YAG laser, wavelength *λ* = 1.064**** *µm*, operating in TEM_00_ mode (Quantronix, East Setauker, NY). The microscope was mounted on a Newport table stabilised against environmental vibrations. Assays were performed using algal slices of approximately 0.2 mm thickness deposited in a 35 mm plastic Petri dish with an adapted glass coverslip at the bottom to observe the samples in high magnification optical microscopy using oil immersion objectives. The samples were moved by step motors adapted to the microscope stage (Prior Scientific, Rockland, MA) with a velocity *V* = 0.91 *µm/s*. The velocity was chosen based on the work of Pontes et al., considering that using slow velocities, up to about 1 micron/sec, the experiments lead to the same results of force measurement [31]. Images of all procedures were recorded with a C2400 charge-coupled device camera (Hamamatsu, Hamamatsu City, Japan) and digitised with an FG7 frame grabber (Scion, Torrance, CA). The image analyses were performed using the software ImageJ, and the data were analysed with KaleidaGraph (Sinergy Software, Essex Junction, VT). After the analysis, the images were processed with ImageJ software (details in “Digital processing of optical microscopy images”).

In each assay, to characterise the adhesion of the *CC* to the algal cell, one cell was chosen, and the sample was positioned in such a way that the laser spot arrived at the centre of one of the *CC* that was at the focus of the optical system. Then, the sample was moved with velocity *V* along one of the two perpendicular directions *x* and *y* in the image plane. For each *CC*, experiments along the two axes were performed. The number of assays, *N,* performed for each treatment was greater than or equal to 10. The following three conditions were considered: 1) *control*, for experiments performed with algae that were not treated with any drug; 2) *colchicine*, for experiments performed with algae treated with colchicine at 0.15 and 1.5 mM for 4 h; and 3) *latrunculin*, for experiments performed with algae treated with latrunculin at 0.1 and 1 µM for 3 days. The statistical analyses were performed with Prism software (Graphpad Software, La Jolla, CA).

**Figure 1:**
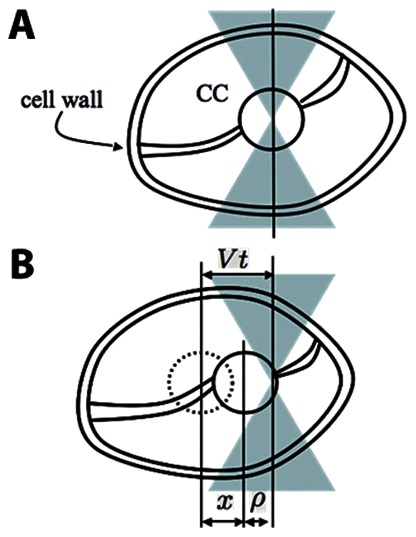
Schematic representation of the experiment with optical tweezers. **a)** *CC* in the equilibrium position of the OT. **b)** When the sample moves with velocity *V*, the alga cell moves accordingly with the same velocity. There appears to be a tug of war between the cell and the OT, as the *CC* changes its equilibrium position in the trap by an amount characterised by *ρ*_,_ and the structures that attach the *CC* to the cell wall exhibit a deformation given by *x*; *t* is the time interval in relation to the beginning of the experiment.

A schematic representation of the experiment is presented in Figure 1. The straight line represents the equilibrium position of the *CC* in the OT. When the sample moves with velocity *V* (Fig. 1b), the alga cell moves accordingly with the same velocity.

There appears to be a tug of war between the cell and the OT when the *CC* changes its equilibrium position in the trap by an amount characterised by *ρ*, and the structures that attach the *CC* to the cell wall has a deformation given by *x*.*t* is the time interval in relation to the beginning of the experiment. The optical force *F_o_* acting on the *CC* is given by
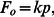
(1)where *k* is the OT stiffness. The force exerted by the cell *F_c_* in the *CC* may be written as
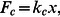
(2)where *k_c_* is defined as the *CC* attachment stiffness. In the whole experiment, the optical force is equivalent to the cell force, *F_o_* = *F_c_*. Moreover, the sample displacement is equal to the change in the equilibrium position of the *CC* in the OT added to the deformation of the structure that attaches the *CC* to the cell wall



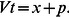
(3)Using Equations 1, 2 and 3, the following relationship is found:
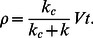
(4)


The dimensionless parameter *A* is defined as
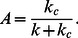
(5)


Because the sample velocity *V* was known, the time interval (*t_max_*) and the maximum trap change in equilibrium position before the *CC* escapes the OT (*ρ_max_*) were measured for each experiment, enabling the determination of *A*

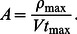
(6)


The optical trap stiffness could not be determined in these experiments. As a result, for each situation analysed, only the ratio 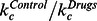 could be determined. From Equation 6, the strength of the *CC* adhesion to the alga cell in the *Control* situation compared to the *Drug* treatment was defined by
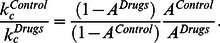
(7)


Thus, considering that *CC* are attached to the cell by a “spring”, the stiffer the spring, the lower the deformation suffered when a given force is applied. Therefore, a higher value of “K” indicates either a stiffer spring or that the *CC* is more strongly attached to the cell periphery.

### Digital Processing of Optical Microscopy Images

Images from optical microscopy were processed with ImageJ software [30] by applying a low pass filter. Thereafter, to obtain the final processed image, a *background subtraction* procedure was performed between the original images and the corresponding image that had been subjected to *low pass filtering*.

### Transcriptomic Analysis

Specimens of *L*. *dendroidea* were collected at Azedinha beach (22°44′28.50″S, 41°52′55.65″W) and Forno beach (22°45′42.49″S, 41°52′29.51″W) in Buzios, and at Ibicuí beach (22°57′44.95″S, 41°01′29.38″W) located in Mangaratiba, on the coast of Rio de Janeiro, Brazil. No specific permissions were required for collection of *L. dendroidea* specimens. The locations are not privately-owned or protected in any way and the field studies did not involve endangered or protected species. The epiphytic biomass was rapidly removed from the collected thalli of *L*. *dendroidea* using tweezers, without damaging the seaweed, and the thalli were immediately frozen in liquid nitrogen. Two specimens from each sample site were separately ground in liquid nitrogen using a mortar and pestle until a fine powder was obtained. One hundred milligrams of powder from each sample was suspended in 1 mL of extraction buffer (6.5 M guanidinium hydrochloride, 100 mM Tris-HCl pH 8.0, 100 mM sodium acetate pH 5.5, 100 mM β-mercaptoethanol, 200 mM KOAc). Total RNA was extracted following the method proposed by Falcão et al. [32], but we performed an extra centrifugation step and transferred the supernatant phase before adding the chloroform, which improved the RNA quality. To eliminate residual DNA, all the samples were treated with DNAse (RNAse free, Promega, Madison, USA). Double-stranded cDNA (ds cDNA) was synthesised and amplified using the SMARTer cDNA synthesis kit and the Advantage 2 polymerase (Clontech, California, USA) starting from 1 µg of total RNA. The optimal number of amplification cycles was determined to be 23. The PCR amplification products were purified using the NucleoSpin® Extract II kit (Macherey-Nagel, Düren, Germany). Finally, the ds cDNA was eluted in TE buffer (Tris-HCl, 10 mM, pH 7.6; EDTA, 1 mM) and sequenced using 454 pyrosequencing technology [33]. The sequences from each sample were preprocessed using Prinseq software [34] to trim poly-A/T tails at least 20 bp long and to remove reads shorter than 75 bp, and then they were assembled into contigs using Roche's algorithm Newbler (minimum overlap length = 40 bp, minimum overlap identity = 95%). In our analysis, we annotated both contigs and singlets after assembly because they contained different sequences and relevant information. The sequences were automatically annotated using the MG-RAST server [35] through BLAST against the COG and KEGG databases with a maximum e-value cut-off of 10^−5^.

## Results

**Figure 2:**
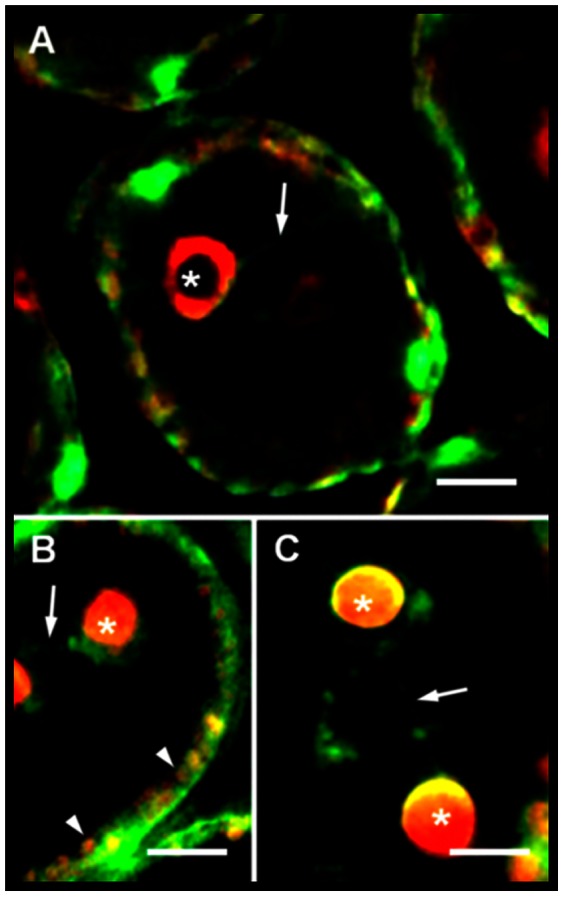
Actin labelling using phalloidin-FITC (A–C). Microfilaments are represented in green and in red-orange the auto-fluorescence of *CC* and chloroplasts. Note that microfilament labelling is not restricted to connection structures (A,C) placed between the *CC* and the cell periphery; the *CC* (A–C) and chloroplasts (B) are also involved in a thin mesh composed of actin filaments. Intercellular connections are also labelled with phalloidin-FITC (A), revealing the presence of actin close to these regions. The asterisks indicate the *CC*; arrows, the membranous tubular connections; and the arrowheads, the chloroplasts. Bars = 10 µm.

In control algal samples, phalloidin-FITC labelling was observed along the cell periphery (Figs. 2 A–B and Figs. S1 A–C), within membranous tubular connections (Figs. 2 A,C and Figs. S1 A–C), and surrounding the *CC* (Figs. 2 A–C and Figs. S1 A–C) and the chloroplasts (Figs. 2 B and Figs. S1 A–C). The labelling of actin microfilaments was diffuse, and longer microfilaments were not observed, whereas smaller fragments appeared more frequently, forming a dense plot that spread in the same regions noted above (Fig. 2 and Figs. S1 A–C). When algal samples were incubated with Lat, the membranous tubular connections linking the *CC* to the cell periphery were disrupted and no actin labelling was observed at this regions or surrounding the *CC* (Fig. S1 D). At cell periphery, the actin labelling surrounding the chloroplasts was weaker than that one observed in untreated algae cells (Fig. S1 D).

**Figure 3:**
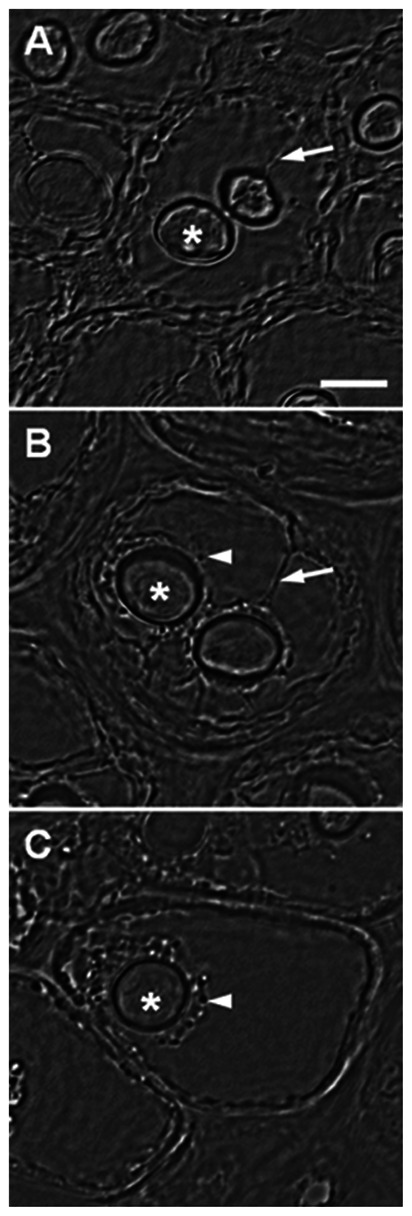
Alga cells treated with latrunculin B. (A) Control, (B) Lat 0.1 µM, (C) Lat 1 µM. * = *CC*, arrows = membranous connections, arrowheads = vesicles. Note the gradual vesicle accumulation surrounding the *CC* (B,C) and the disruption of connective structures between the *CC* and the cell periphery (C). Bar = 9 µm.

Using the bright field microscopy, it could be observed that the Lat treatment has induced changes in the cell structure that were positively correlated with the increased Lat concentration (Fig. 3 and Table S1). The membranous tubular connections linking the *CC* to the cell periphery were disrupted, and therefore the traffic of vesicles was interrupted (Fig. 3 C and Table S1). Due to this interruption, a cluster of vesicles was observed surrounding the *CC*, mainly in algae subjected to higher concentrations of Lat (Fig. 3 C and Table S1).

**Figure 4:**
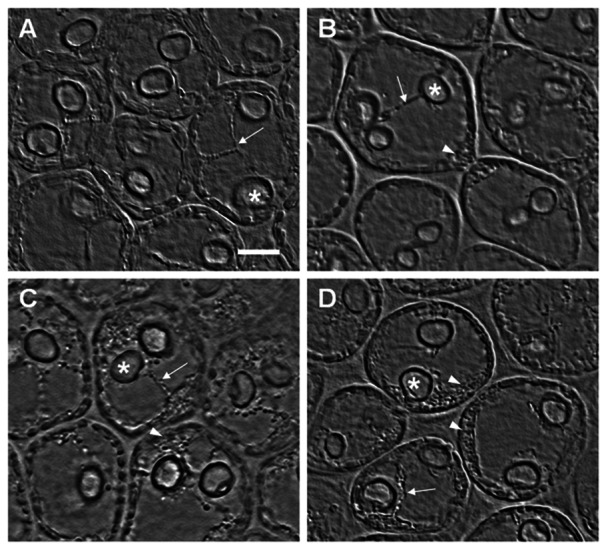
Alga cells treated with colchicine. (A) Control, (B) 0.15 mM colchicine, (C, D) 1.5 mM colchicine.* = *CC*, arrows = connections structures, arrowheads = vesicles. Note the progressive accumulation of vesicles in the cell periphery (C, D) and the high accumulation of vesicles close to intercellular connections (B,D). Bar = 10 µm.

As observed in the Lat-treated algae, the colchicine treatment showed a trend towards a higher level of filament disorganisation with increased drug concentration. However, the membrane tubular connections were not disrupted (Fig. 4 A–D). The microtubule destabilisation induced by colchicine did not significantly change the structure of the connections (Fig. 4 A–D), and the main effect observed was an increase in the number of vesicles within the cell as the drug concentration increased (Figs. 4 B–D and Table S1). After treatment with the lower colchicine concentration, the vesicle accumulation occurred in the cell periphery near cell wall regions resembling intercellular connections (Fig. 4 B and Table S1). Treatment with the higher colchicine concentration caused more vesicle accumulation, mainly in the cell periphery (Figs. 4 C–D and Table S1).

In relation to the assay from double-treated algae (incubation with Lat and colchicine), it was observed a result similar to the latrunculin assay, where the vesicle traffic was interrupted and the vesicles accumulated in regions surrounding the CC (Figure S2 and Table S1).

**Figure 5:**
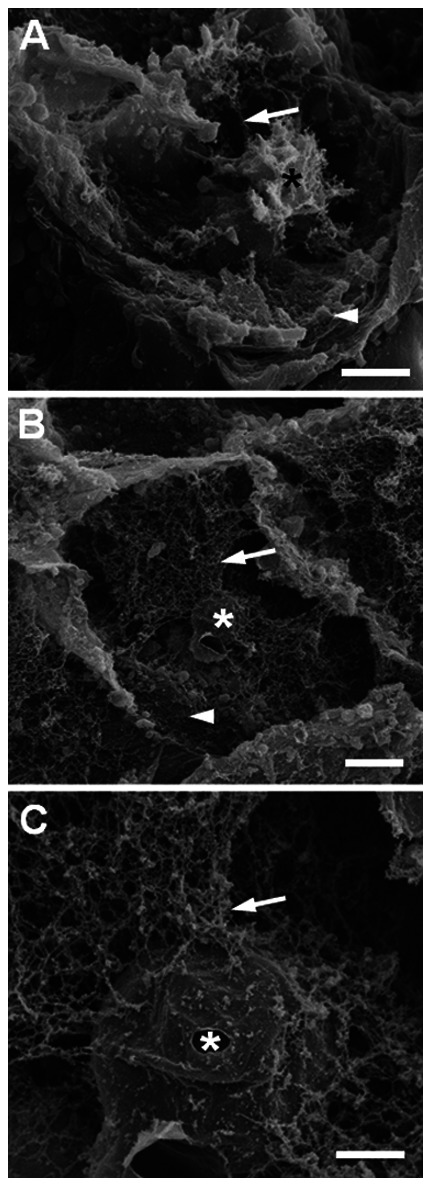
Alga cells treated with paclitaxel. (A) Control, (B,C) 0.12 mM paclitaxel. * = *CC*, arrow = cytoskeleton filaments, arrowheads = vesicles. Few vesicles are observed dispersed in the cell periphery, in cytoskeletal filaments surrounding the *CC* or forming connections from the *CC* to the cell periphery. In cells treated with paclitaxel, note that larger connective structures were formed linking the *CC* to the cell walls. Bars = 10 µm (A,B) and 2 µm (C).

Fracturing of the *L. dendroidea* thallus allowed for observation, by scanning electron microscopy, of the inner space of cortical cells (Fig. 5) and also of some membranous compartments, such as the membranous tubular connections and the main vacuolar space (Fig. 5). In untreated cells (control), we observed the following characteristics: the *CC* in the central cell region, few vesicles dispersed in the cell periphery, and cytoskeletal filaments surrounding the *CC* or forming connections from the *CC* to the cell periphery (Fig. 5A). The algae treated with paclitaxel exhibited a dense cytoskeletal network, mainly adhered to cell periphery and to the *CC* (Figs. 5 B–C). Large connective structures linking the *CC* to the cell wall and some vesicles attached to the cell wall were observed (Fig. 5 B). Many cytoskeletal filaments were bound to the *CC* (Fig. 5 C).

**Figure 6:**
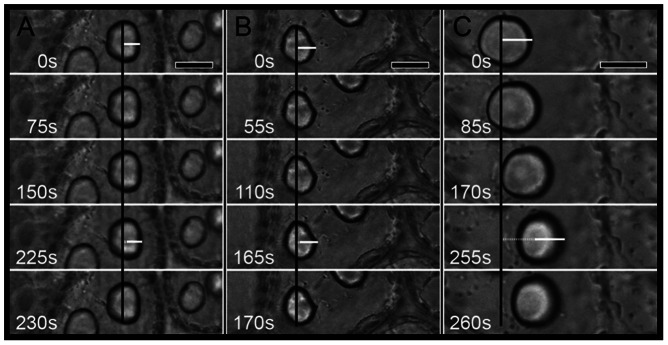
*Corps en cerise* manipulation with optical tweezers during microscopy. Control cells (A), cells from algae treated with 1 µM Lat (B) and cells from algae treated with 1.5 mM colchicine (C). The black line marks the initial *CC* position, and the dashed white lines mark the *CC* displacement. Note that no apparent displacement was observed in the cells from the control and Lat treatments, whereas in the colchicine treatment, the *CC* was displaced more easily, indicating that the *CC* is weakly adhered to the cell body when cells are treated with 1.5 mM colchicine. Bars = 8 µm.

The use of optical tweezers microscopy allowed for the determination of active cytoskeletal filaments in the process of anchoring the *CC* to the cell periphery (Table 1 and Fig. 6). The latrunculin treatments were not effective in decreasing the binding force of the *CC* to the cell periphery (Table 1 and Fig. 6B). In contrast, treatment with a high concentration of colchicine significantly modified the adhesion strength of the *CC* to the cell periphery (Table 1), resulting in an improved ability to handle the *CC* with optical tweezers (Fig. 6 C).

**Table 1:** Mean values of the ratio between *K^control^* and *K^drugs^* (latrunculin and colchicine).

Drug treatment	Values of the ratio *K^control^/K^drugs^*
Latrunculin [0.1 µM]	1.1±0.2
Latrunculin [1 µM]	1.0±0.2
Colchicine [0.15 mM]	1.3±0.2
Colchicine [1.5 mM]	1.8±0.3*

Standard errors are given after the mean values. The asterisk indicates a significant difference between the control and drug treatment (p<0.001).

The transcriptomic analysis revealed the expression of genes related to cytoskeletal activity and to intracellular trafficking (Tables 2 and 3). Among these genes, the expression of actin, tubulin and associated proteins, such as dynein light chain, tropomyosin 1, alpha-actinin, actin bundling proteins fimbrin/plastin (Table 2), kinesin-like protein and myosin heavy chain (the latter two from not assembled sequences),was observed. With regard to vesicle traffic (Table 3), we confirmed the expression of genes related to vesicle budding, vesicle sorting and fusion, secretory activity and endocytic activity. In addition, we identified genes related to Golgi activity, transport from the ER to the Golgi complex and retrograde transport. Other related cell signalling transcripts were identified along with transcripts from the PI-3 kinase and Ca^2+^/CaM-dependent kinase families.

**Table 2:** Main transcripts related to cytoskeleton in *L. dendroidea*.

	R.A. (%)	Database
*Main components of cytoskeleton*		
Tubulin related proteins	1.15	COG
Actin related proteins	1.07	KEGG
Actin beta/gamma 1	1.07	KEGG
Tubulin alpha	0.73	KEGG
Tubulin beta	0.92	KEGG
*Cytoskeleton associated proteins*		
Tropomyosin 1	0.10	KEGG
Dynein light chain LC8-type	0.10	COG
Actinin alpha	0.05	KEGG
Ca^++^-binding actin-bundling protein fimbrin/plastin (EF-Hand superfamily)	0.04	COG
*Cytoskeleton regulation and dynamics*		
Phosphatidylinositol kinase and protein kinases of the PI-3 kinase family	0.36	COG
Calcium/calmodulin-dependent protein kinase	0.05	KEGG

The relative abundance (R.A.) of transcripts detected in samples from three populations of *L. dendroidea* and the database used for sequence annotation are shown.

**Table 3:** Main transcripts related to intracellular trafficking, secretion and vesicular transport in *L. dendroidea*.

	R.A. (%)	Database
*Vesicle budding*		
Clathrin adaptor complex, small subunit	0.20	COG
Vesicle coat complex COPII, subunit SEC23	0.16	COG
Vesicle coat complex, various subunits	0.12	COG
Charged multivesicular body protein 1	0.05	KEGG
*Vesicle sorting and fusion*		
Vesicle-fusing ATPase	0.14	KEGG
Synaptobrevin/VAMP-like protein	0.12	COG
Synaptobrevin homolog YKT6	0.10	KEGG
T-SNARE complex subunit, syntaxin	0.04	COG
Syntaxin 16	0.05	KEGG
Syntaxin 5	0.05	KEGG
Vesicle transport protein SEC22	0.05	KEGG
*Secretory pathway*		
Ras-related protein Rab-11A	0.05	KEGG
*Endocytosis*		
Phosphatidylinositol kinase and protein kinases of the PI-3 kinase family	0.36	COG
ESCRT-II complex subunit VPS22	0.05	KEGG
Clathrin adaptor complex, small subunit	0.20	COG
Charged multivesicular body protein 1	0.05	KEGG
*Vacuolar activity*		
Vacuolar protein-sorting-associated protein 4	0.10	KEGG
Protein involved in vacuole import and degradation	0.04	COG
*Golgi activity*		
Membrane protein involved in Golgi transport	0.04	COG
Peripheral Golgi membrane protein	0.04	COG
*Retrieval transport to ER*		
ER lumen protein retaining receptor	0.04	COG
Golgi protein involved in Golgi-to-ER retrieval	0.04	COG

The relative abundance (R.A.) of transcripts detected in samples from three populations of *L. dendroidea* and the database used for sequence annotation are shown.

## Discussion

In plants, secondary metabolite transport is usually attributed to specific membrane proteins, such as ABC transporters [36]. However, due to the lipophilic nature of some secondary metabolites, it has been suggested that the intra-cellular trafficking of these substances can also be accomplished by vesicles [36]. Indeed, the partitioning of secondary metabolites into vesicles is an event that is frequently observed in macroalgae [16]–[19], [37]–[38]. Physodes, for example, are organelles in which phenolic compounds are packaged in brown algae, and they are found in many macroalga species [39]–[44]. In brown macroalgae, the transport of phenolic compounds is a key event in wall formation and is dependent on the movement of physodes, which occurs along cytoskeletal filaments [24]. The actin cytoskeleton functions as a general circulatory system, moving physodes through the cell, and microtubules play a role in the specific traffic of phenols to regions near the plasma membrane where exocytosis will occur [24].

In red algae, however, there has not been a study relating the cytoskeleton behaviour with the traffic of secondary metabolites and chemical defence. Relatively few studies have contributed to understanding the mechanism of intra-cellular secondary metabolite transport in red algae [16], [17], [19]–[21]. Although some studies have noted the *gland cells* as structures that are specialised for the synthesis and excretion of secondary metabolites [16]–[17], there is no strong evidence confirming the existence of a vesicle-mediated transport in these cells. As for species in the genus the *Laurencia*, the storage of halogenated compounds within the *corps en cerise* organelle [19], [21] and the vesicle transport of halogenated metabolites to the cell walls has been demonstrated [19]–[21].

With regard to cytoskeletal studies in red algae, many reports have used stabilising and destabilising drugs to interfere with the normal dynamics of actin microfilaments and microtubules. To study the actin cytoskeleton, potassium iodide, cytochalasin-B, latrunculin-A [45], cytochalasin D, latrunculin B and the myosin inhibitor butanedionemonoxime [46] have been used. By using latrunculin B, for example, the participation of actin filaments in pseudopodia formation of *Porphyra pulchella* was shown [46]. In that study, the authors used latrunculin B at 12.5 µM, a concentration more than tenfold higher than the highest concentration used in our experimental conditions, which was 1 µM. Drugs that interfere with the dynamics of cytoskeletal filaments have been used in different concentrations. Even low concentrations of latrunculin B (from 0.05 µM to 1 µM) are effective, as observed in root cells of *Arabidopsis* [47]. These different latrunculin effective concentrations may be possible caused by different actin filament properties (such filaments length), or different permeability of cell wall and cell membrane or different experimental conditions. In our work, higher latrunculin concentrations (over 5 µM) were lethal to *L. dendroidea*, causing cells death (data not shown).

The treatments with 0.1 and 1 µM of latrunculin B indicated the presence of microfilaments in membranous tubular structures due to the lack of integrity of these structures, which connect the *CC* to the cell periphery. Vesicles budding from the *CC* were not interrupted by this treatment, and as a consequence, they accumulated around the *CC*. The presence of microfilaments at the connection structures was confirmed by fluorescently labelled phalloidin staining. Taken together, these results indicate that actin filaments are involved in the traffic of vesicles containing secondary metabolites from the *CC* to the cell periphery in *L. dendroidea*.

The microtubule network is also related to the normal traffic of vesicles from the *CC*. Specifically, we demonstrated that the transport of vesicles at the cell periphery is dependent on microtubule activity, which was interrupted due to the colchicine treatment. In *L. dendroidea*, this microtubule-depolymerising drug caused the accumulation of many vesicles at this region, indicating the participation of microtubules in vesicle sorting and positioning near the plasma membrane. In addition, the algae treated with both Lat and colchicine drugs also presented the vesicles accumulation close to the *CC*. Since no vesicle accumulation was seen in cell periphery, even with the colchicine treatment, this result reveals a decrease in the sorting of vesicles arising from the membranous tubular structures. This result confirms the inhibition of vesicle traffic near the CC, caused by the latrunculin treatment, and also confirms that vesicles accumulated in cell periphery comes from the CC (with only colchicine treatment). Taken together, these results indicates that the actin filaments play an essential role in vesicle traffic along the connective tubular structures while the microtubules are involved in the vesicle transport at cell periphery.

In contrast, in the unicellular red alga *Glaucosphaera vacuolata*, the microtubules perform the transport of vesicles from the central region to the cell periphery, and the actin cytoskeleton is peripherally distributed and appears to be involved in vesicle exocytosis [25]. The cytoskeletal activity in *L. dendroidea* was similar to that observed in plants [48], where, although the traffic of membrane vesicles is generally dependent on actin filaments and myosin motors, the movement and positioning involves interactions with microtubules [48]. The actin-myosin system provides motility in plants, and microtubules appear to stabilise the positioning of organelles, most likely involving specific kinesins [48].

Interestingly, algae treated with lower colchicine concentrations presented a discrete pattern of vesicle distribution, most likely reflecting the early stage of microtubule destabilisation. This pattern reveals a preferential routing of vesicles to areas where cell-to-cell communication is found, reflecting a possible strategy for tissue preservation in red macroalgae. This result seems to indicate that once the microtubule activity was affected, some specific target vesicles were transported by actin filaments. Previous studies reported a similar event in plant cells, where cell-to-cell communication utilises the actin cytoskeleton as a shuttle for the focal accumulation of defence responses at the cell surface adjacent to dead or dying cells, extending beyond the conventional role of actin defence in infected cells to a prophylactic role in protecting the surrounding healthy cells [49]. In addition, in mammalian immune cells, focal secretion of toxic substances against malignant target cells, i.e., the immunological synapses, occurs in cytotoxic T cells and depends on comprehensive cytoskeletal reorganisation [50].

In addition to their role in vesicle traffic and chemical defence, microtubules are crucial to the correct positioning and anchoring of the *CC* at the cell periphery, as shown by the use of optical tweezers. The destabilisation of these filaments after colchicine treatment allowed for the effortless manipulation of the *CC* organelle with optical tweezers. However, in control and latrunculin B-treated algae, the *CC* was not easily moved within the cell space. Although few studies have used optical tweezers to manipulate organelles, this tool has proven to be useful for better understanding the interactions between organelles and the cell as a whole, including its cytoskeletal network. In *Spinacia oleraceae*, the use of optical tweezers allowed for the study of cytoskeletal interactions with organelles, confirming the presence of a mesh connecting nearby chloroplasts to each other and indicating the participation of fine cytoplasmic strands or microfilaments in this process [51]. In the soybean *Glycine max*, the tension of cytoplasmic strands was determined with optical tweezers, and, during the application of 20 µM cytochalasin D, the tension in these strands markedly decreased, confirming the presence of actin microfilaments in the composition of the cytoplasmic strands [52]. Optical tweezers were also employed to investigate the role of the actin cytoskeleton in regulating cytoplasmic stiffness [52], [53], which is reduced after treatment with an actin-depolymerising agent [53]. In the present study, we confirmed that microtubules are involved in organelle positioning, specifically CC positioning, with no evident participation of the actin cytoskeleton in this process. The results obtained by scanning electron microscopy also corroborate the hypothesis that microtubules are involved in the anchoring and positioning of the *CC*. After treatment with paclitaxel, the cytoskeletal network appears as a dense and thick mesh connecting the *CC* to the cell periphery and is morphologically distinct from the thin connective structures where vesicle traffic takes place.

The transcriptomic analysis presented in this study constitutes the first characterisation of the genes involved in cytoskeleton and vesicle traffic in the red seaweed genus *Laurencia*. Moreover, this approach, in conjunction with the cellular assays, expands the knowledge of the intracellular transport of defensive compounds in *L*. *dendroidea*, a seaweed rich in cytotoxic terpenoid compounds [10]. In comparison with gene expression profiles from other marine macroalgae [54]–[62], the results obtained here represent a comprehensive survey with regard to cytoskeleton and vesicle trafficking, given that more than 30 distinct gene families were recognised. In accordance with these results, the main cytoskeletal transcripts identified in other macroalgae are tubulin and actin; specifically, actin 1 in *Griffithsia okiensis* [54]; actin, tubulin beta subunit and tubulin alpha-2 ? alpha-4 chain in *Porphyra haitanensis* [55]; and at least three different actins in *Gracilaria tenuistipitata* [56]. The first discovery of γ-tubulin in red algae was reported after proteome analysis of *Gracilaria changii* [57]. Cytoskeleton-associated proteins are also present in the expression profiles of other algae, highlighting motor proteins such as myosin heavy chain in *G. changii* [58] and microtubule motor activity in *Ectocarpus siliculosus* [59]. Several studies indicate that the dynamic nature of the cytoskeleton is also reflected in the expression patterns of the genes coding for these proteins, as evidenced by the change in the abundance of transcripts for actin cytoskeletal 1 LPC1 and tubulin alpha-123 under light deprivation in *Gracilaria changii* [60] and the change in the expression levels of actin, alpha tubulin subunit and ARP1 actin-related protein, which have been putatively linked to salinity stress tolerance in *Furcellaria lumbricalis* [61]. Furthermore, the proteomic profile of *Saccharina japonica* revealed a seasonal variation in actin content [62], and the induction of diverse genes related to vesicular trafficking and the cytoskeleton under oxidative stress was observed in *Ectocarpus siliculosus* [59].

In keeping with the results of our cellular analysis, transcriptome sequencing showed motor activity associated with microfilaments and microtubules based on the identification of sequences of dynein light chain, kinesin-like protein and myosin heavy chain. Other accessory proteins were recognised in *L. dendroidea*, but these were mostly transcripts of PI-kinases and Ca^++^/calmodulin-dependent kinases, which may compose a part of the machinery for cytoskeleton and exocytosis regulation [63], [64].

Regarding vesicle trafficking, we obtained an extensive list of putative proteins involved in this process in *L. dendroidea*. Among these proteins, the expression of clathrins and SNAREs have already been related to stress response in *G. changii* and *E. siliculosus*, respectively [59], [60]. We have also identified several transcripts that are associated with vesicle budding, vesicle sorting, vesicle fusion and the secretory pathway (all relevant for secondary metabolite exocytosis). Finally, the identification of genes related to endocytosis, vacuolar activity, Golgi activity and retrieval transport to ER is in agreement with the high rate of vesicle transport activity previously reported in *L. dendroidea* [21]. In agreement with these data, significant activity directed to the recycling of membranes and resident proteins to their donor organelles was shown, which are processes typically observed in cells with high secretory activity.

Here, we showed that the transport of vesicles in *L.dendroidea* depends on both actin microfilaments and microtubules. Specifically, we observed that actin filaments found in membranous tubular connections act to transport vesicles from the *CC*, the main storage site of halogenated compounds, to the cell periphery. In a second step, the microtubules perform an essential role in positioning the vesicles along the cell periphery to specific regions where exocytosis takes place. In addition, microtubules are involved in anchoring and positioning the *CC*. Thus, we demonstrated for the first time that actin microfilaments and microtubules play an essential role in chemical defence in red macroalgae.

## Supporting Information

Figure S1 **LSCM showing the actin labelling with phalloidin-FITC (A–D).** A control cell (latrunculin untreated alga) can be seen in two different focal planes (A,B) and the sum of the focal planes obtained is also shown (C). The figure D corresponds to a latrunculin B treated cell (sum of focal planes obtained). Microfilaments are represented in green and, in red-orange, the auto-fluorescence of *CC* and chloroplasts. In the latrunculin untreated cell (A–C), it is possible to observe the microfilament labelling in connection structures (arrows), over the *CC* region (*) and surrounding the chloroplasts (arrowheads), while in a latrunculin treated cell, the connecting structure was not observed, nor was observed an actin labelling in *CC* region (*). At this treatment, the phalloidin labelling was seen surrounding the chloroplasts (arrowheads), but weaker than the fluorescence observed in untreated cell (control). Bars = 5 µm.
(TIF)

Figure S2 **Alga cells double-treated with cytoskeleton depolymerising drugs (both with 1 µM latrunculin and 1.5 mM colchicine) (A,B).** * = *CC*, arrow = vesicles. Note the vesicle accumulation surrounding the *CC* and the absence of connective structures between the *CC* and the cell periphery (C). Bar = 3 µm.
(TIF)

Table S1 **Mean values of the number of vesicles accumulated in response to different drug treatments (latrunculin, colchicine and latrucunlin+colchicine).** The vesicles accumulation were observed in regions surrounding the CC (in algae cells treated with latrunculin and treated with both drugs simultaneously) and at the cell periphery (in algae cells treated with colchicine). Standard deviations are given after the mean values. The asterisk indicates significant difference between the control and the drug treatments (p<0.01).
(DOCX)

## References

[1] Blunt JW, Copp BR, Keyzers RA, Robert A, Munro MHG, et al (2012) Marine natural products. Nat Prod Rep 29: 144–222. 22193773 10.1039/c2np00090c

[2] Paul VJ, Kuffner IB, Walters LJ, Ritson-Williams R, Beach KS, et al (2011) Chemically mediated interactions between macroalgae *Dictyota* spp. and multiple life-history stages of the coral *Porites astreoides* . Mar Ecol Prog Ser 426: 161–170.

[3] Nylund GM, Gribben PE, De Nys R, Pavia H (2007) Surface chemistry versus whole-cell extracts: antifouling tests with seaweed metabolites. Mar Ecol Prog Ser 329: 73–84.

[4] Bianco EM, Teixeira VL, Pereira RC (2010) Chemical defenses of the tropical marine seaweed *Canistrocarpus cervicornis* against herbivory by sea urchin. Braz J Oceanogr 58: 213–218.

[5] Amsler CD, Fairhead VA (2006) Defensive and sensory chemical ecology of brown algae. Adv Bot Res 43: 1–91.

[6] Erickson KL (1983) Chemical and biological perspectives. In: Scheuer P J, editor. Marine natural products. (ed.), New York: Academic Press, 131–257.

[7] Cassano V, Yola M, Millar AJK, Gil-Rodriguez MC, Senties A, et al (2012) Redefining the taxonomic status of *Laurencia dendroidea* (Ceramiales, Rhodophyta) from Brazil and the Canary Islands. Eur J Phycol 47: 67–81.

[8] Da Gama BAP, Carvalho AGV, Weidner K, Soares AR, Coutinho R, et al (2008) Antifouling activity of natural products from Brazilian seaweeds. Bot Mar 51: 191–201.

[9] Da Gama BAP, Pereira RC, Carvalho AGV, Coutinho R, Yoneshigue-Valentin Y (2002) The effects of seaweed secondary metabolites on biofouling. Biofouling 18: 13–20.

[10] Pereira RC, Da Gama BAP, Teixeira VL, Yoneshigue-Valentin Y (2003) Ecological roles of natural products of the brazilian red seaweed *Laurencia dendroidea* . Braz J Biol 63: 665–672. 15029377 10.1590/s1519-69842003000400013

[11] Amsler CD (2008) Algal chemical ecology. Springer 313p.

[12] Hay ME (2009) Marine chemical ecology: Chemical signals and cues structure marine populations, communities, and ecosystems. Ann Rev Mar Sci 1: 193–212. 10.1146/annurev.marine.010908.163708 PMC3380104 21141035

[13] Paul VJ, Ritson-Williams R, Sharp K (2011) Marine chemical ecology in benthic environments. Nat Prod Rep 28: 345–387. 21125086 10.1039/c0np00040j

[14] Pelletreau KN, Targett NM (2008) New perspectives for addressing patterns of secondary metabolites in marine macroalgae. In: Amsler CD, editor. Algal chemical ecology. New York: Springer. pp. 121–146.

[15] Pereira RC, Da Gama BAP (2008) Macroalgal chemical defenses and their roles in structuring tropical marine communities. In: Amsler CD, editor., Algal chemical ecology. New York: Springer. pp. 25–55.

[16] Paul NA, Cole L, De Nys R, Steinberg PD (2006) Ultrastructure of the gland cells of the red alga *Asparagopsis armata* (Bonnemaisoniaceae). J Phycol 42: 637–645.

[17] Dworjanyn SA, De Nys R, Steinberg PD (1999) Localisation and surface quantification of secondary metabolites in the red alga *Delisea pulchra* . Mar Biol 133: 727–736.

[18] Young DN, Howard BN, Fenical W (1980) Subcellular localization of brominated secondary metabolites in the red alga *Laurencia snyderae.* J Phycol 16 182–185.

[19] Salgado LT, Viana NB, Andrade LR, Leal RN, Da Gama BAP, et al (2008) Intra-cellular storage, transport and exocytosis of halogenated compounds in marine red alga *Laurencia dendroidea* . J Struct Biol 162: 345–355. 18337120 10.1016/j.jsb.2008.01.015

[20] Sudatti DB, Rodrigues SV, Coutinho R, Da Gama BAP, Salgado LT, et al (2008) Transport and defensive role of elatol at the surface of the red seaweed *Laurencia dendroidea* (Ceramiales, Rhodophyta). J Phycol 44: 584–591. 27041418 10.1111/j.1529-8817.2008.00507.x

[21] Paradas WC, Salgado LT, Sudatti DB, Crapez MA, Fujii MT, et al (2010) Induction of halogenated vesicle transport in cells of the red seaweed *Laurencia obtusa* . Biofouling 26: 277–286. 10.1080/08927010903515122 20077237

[22] Steinberg PD, De Nys R (2002) Chemical mediation of colonization of seaweeds surfaces. J Phycol 38: 621–629.

[23] Lane AL, Nyadong L, Galhena AS, Shearer TL, Stout EP, et al (2009) Desorption electrospray ionization mass spectrometry reveals surface-mediated antifungal chemical defense of a tropical seaweed. Proc Natl Acad Sci USA 106: 7314–7319. 19366672 10.1073/pnas.0812020106 PMC2678663

[24] Schoenwaelder MEA, Clayton MN (1999) The role of the cytoskeleton in brown alga physode movement. Eur J Phycol 34: 223–229.

[25] Wilson SM, Pickett-Heaps JD, West JA (2006) Vesicle transport and the cytoskeleton in the unicellular red alga *Glaucosphaera vacuolata.* . Phycol Res 54: 15–20.

[26] Russell CA, Guiry MD, McDonald AR, Garbary DJ (1996) Actin-mediated chloroplast movement in *Griffıthsia pacifica* (Ceramiales, Rhodophyta). Phycol Res 44: 57–61.

[27] Von Stosch H (1963) Wirkungen von Jod und Arsenit auf Meeresalgen in Kultur, In: De Virville D, Feldmann J, editors. Proceedings of the Fourth International Seaweed Symposium. Oxford: Pergamon Press. pp. 142–150.

[28] Lewin J (1966) Silicon metabolism in diatoms. V. Germanium dioxide, an especific inhibitor of diatom growth. Phycologia 6: 1–12.

[29] Oliveira EO, Paula EJ, Plastino EM, Petti R (1995) Metodologias para cultivo no axênico de macroalgas marinas in vitro, In: Alveal K, Ferrario ME, Oliveira EC, Sar E, editors. Manual de métodos ficológicos. Chile: Universidad de Concepción. pp. 430–447.

[30] Abramoff MD, Magalhães PJ, Ram SJ (2004) Image processing with ImageJ. J Biophotonics 11: 36–42.

[31] Pontes B, Viana NB, Salgado LT, Farina M, Moura Neto M, et al (2011) Cell cytoskeleton and tether extraction. Biophys J 101: 43–52. 21723813 10.1016/j.bpj.2011.05.044 PMC3127177

[32] Falcão VR, Tonon AP, Oliveira MC, Colepicolo P (2008) RNA Isolation method for polysaccharide rich algae: Agar producing *Gracilaria tenuistipitata* (Rhodophyta). J Appl Phycol 20: 9–12.

[33] Margulies M, Egholm M, Altman WE, Attiya S, Bader JS, et al (2005) Genome sequencing in microfabricated high-density picolitre reactors Nature. 437: 376–380. 10.1038/nature03959 PMC1464427 16056220

[34] Schmieder R, Edwards R (2011) Quality control and preprocessing of metagenomic datasets. Bioinformatics 27: 863–864. 21278185 10.1093/bioinformatics/btr026 PMC3051327

[35] Meyer F, Paarmann D, D'Souza M, Olson R, Glass EM, et al (2008) The metagenomics RAST server - a public resource for the automatic phylogenetic and functional analysis of metagenomes. BMC Bioinformatics 9: 386–393. 18803844 10.1186/1471-2105-9-386 PMC2563014

[36] Yazaki K (2006) ABC transporters involved in the transport of plant secondary metabolites. FEBS Lett 580: 1183–1191. 10.1016/j.febslet.2005.12.009 16364309

[37] Manning SR, La Claire JW (2010) Prymnesins: Toxic metabolites of the golden alga, *Prymnesium parvum* Carter (Haptophyta). Mar Drugs 8: 978–704. 10.3390/md8030678 PMC2857367 20411121

[38] Ragan MA (1976) Physodes and the phenolic compounds of brown algae. Composition and significance of physodes *in vivo* . Botanica Marina 19: 145–154.

[39] Salgado LT, Andrade LR, Amado-Filho GM (2005) Localization of specific monosaccharides in cells of the brown alga *Padina gymnospora* and the relation to heavy-metal accumulation. Protoplasma 225: 123–128. 15868219 10.1007/s00709-004-0066-2

[40] Kaur I, Vijayaraghavan MR (1992) Physode distribution and genesis in *Sargassum vulgare* C. Agardh and *Sargassum johnstonii* Setchell & Gardner. Aquat Bot 42: 375–384.

[41] Schoenwaelder MEA, Wiencke C (2000) Phenolic compounds in the embryo development of several northern hemisphere Fucoids. Plant Biol 2: 24–33.

[42] Lüder UH, Clayton MN (2004) Induction of phlorotannins in the brown macroalga *Ecklonia radiata* (Laminariales, Phaeophyta) in response to simulated herbivory–the first microscopic study. Planta 218: 928–937. 14716562 10.1007/s00425-003-1176-3

[43] Pellegrini L (1980) Cytological studies on physodes in the vegetative cells of *Cystoseira stricta* Savageau (Phaeophyta, Fucales) J Cell Sci. 41: 209–231. 10.1242/jcs.41.1.209 7364883

[44] Brawley SH (1976) Fine-structural studies of the gametes and embryo of *Fucus vesiculosus* L. (Phaeophyta), J Cell Sci. 20: 255–271. 10.1242/jcs.20.2.255 944190

[45] Kim SH, Kim GH (1999) The role of F-actin during fertilization in the red alga *Aglaothamnion oosumiense* (Rhodophyta) J Phycol. 35: 806–814.

[46] Ackland JC, West JA, Pickett-Heaps J (2007) Actin and myosin regulate pseudopodia of *Porphyra pulchella* (Rhodophyta) archeospores. J Phycol 43: 129–138.

[47] Hou G, Kramer VL, Wang YS, Chen R, Perbal G, et al (2004) The promotion of gravitropism in *Arabidopsis* roots upon actin disruption is coupled with the extended alkalinization of the columella cytoplasm and a persistent lateral auxin gradient. Plant J 39: 113–125. 15200646 10.1111/j.1365-313X.2004.02114.x

[48] Petrášek J, Schwarzerová K (2009) Actin and microtubule cytoskeleton interactions. Curr Opin Plant Biol 12: 728–734. 19854097 10.1016/j.pbi.2009.09.010

[49] Day B, Henty JL, Porter KJ, Staiger CJ (2011) The Pathogen-Actin Connection: A Platform for Defense Signaling in Plants. Phytopathology 49: 483–506. 10.1146/annurev-phyto-072910-095426 21495845

[50] Schmidt SM, Panstruga R (2007) Cytoskeleton functions in plant–microbe interactions. Physiol Mol Plant Path 71: 135–148.

[51] Bayoudh S, Mehta M, Rubinsztein-Dunlop H, Heckenberg NR, Critchley C (2001) Micromanipulation of chloroplasts using optical tweezers. J Microsc 203: 214–222. 11489079 10.1046/j.1365-2818.2001.00843.x

[52] Grabski S, Xie XG, Holland JE, Schindler M (1994) Lipids Trigger Changes in the Elasticity of the Cytoskeleton in Plant Cells: A Cell Optical Displacement Assay for Live Cell Measurements. J Cell Biol 126: 713–726. 8045935 10.1083/jcb.126.3.713 PMC2120140

[53] Hawes C, Osterrieder A, Sparkes IA, Ketelaar T (2010) Optical tweezers for the micromanipulation of plant cytoplasm and organelles. Curr Opin Plant Biol 13: 731–735. 21093352 10.1016/j.pbi.2010.10.004

[54] Lee H, Lee HK, An G, Lee YK (2007) Analysis of Expressed Sequence Tags from the Red Alga *Griffithsia okiensis* . J Microbiol 45: 541–546. 18176538

[55] Xiaolei F, Yongjun F, Songnian H, Guangce W (2007) Generation and analysis of 5318 expressed sequence tags from the filamentous sporophyte of *Porphyra haitanensis* (Rhodophyta). J Phycol 43: 1287–1294.

[56] Collén PN, Collén J, Da Silva Reis M, Pedersén M, Setubal JC, et al (2012) Analysis of expressed sequence tags from the agarophyte *Gracilaria tenuistipitata* (Rhodophyta). J Appl Phycol 24: 641–647.

[57] Wong PF, Tan LJ, Nawi H, Abu Bakar S (2006) Proteomics of the red alga, *Gracilaria changii* (Gracilariales, Rhodophyta). J Phycol 42: 113–120.

[58] Teo SS, Ho CL, Teoh S, Lee WW, Tee JM, et al (2007) Analyses of expressed sequence tags from an agarophyte, *Gracilaria changii* (Gracilariales, Rhodophyta).Eur J Phycol. 42: 41–46.

[59] Dittami SM, Scornet D, Petit JL, Ségurens B, Da Silva C, et al (2009) Global expression analysis of the brown alga *Ectocarpus siliculosus* (Phaeophyceae) reveals large-scale reprogramming of the transcriptome in response to abiotic stress. Genome Biol 10: R66. 19531237 10.1186/gb-2009-10-6-r66 PMC2718500

[60] Ho CL, Teo HS, Teo SS, Rahim RA, Phang SM (2009) Profiling the transcriptome of *Gracilaria changii* (Rhodophyta) in response to light deprivation. Mar Biotechnol 11: 513–519. 19043658 10.1007/s10126-008-9166-x

[61] Kostamo K, Olsson S, Korpelainen H (2011) Search for stress-responsive genes in the red alga *Furcellaria lumbricalis* (Rhodophyta) by expressed sequence tag analysis. J Exp Mar Biol Ecol 404: 21–25.

[62] Yotsukura N, Nagai K, Kimura H, Morimoto K (2010) Seasonal changes in proteomic profiles of Japanese kelp: *Saccharina japonica* (Laminariales, Phaeophyceae),J Appl Phycol. 22: 443–451.

[63] Žárský V, Cvrcková F, Potocký M, Hála M (2009) Exocytosis and cell polarity in plants - exocyst and recycling domains. New Phytol 183: 255–272. 19496948 10.1111/j.1469-8137.2009.02880.x

[64] Li L, Saga N, Mikami K (2008) Phosphatidylinositol 3-kinase activity and asymmetrical accumulation of F-actin are necessary for establishment of cell polarity in the early development of monospores from the marine red alga *Porphyra yezoensis* . J Exp Bot 59: 3575–3586. 18703492 10.1093/jxb/ern207 PMC2561153

