# Supplementary figures and images for: Traffic of Secondary Metabolites to Cell Surface in the Red Alga Laurencia dendroidea Depends on a Two-Step Transport by the Cytoskeleton
Source: PLoS One. 2013 May 21;8(5):e63929. doi: 10.1371/journal.pone.0063929 (PMC3660350; doi:10.1371/journal.pone.0063929)

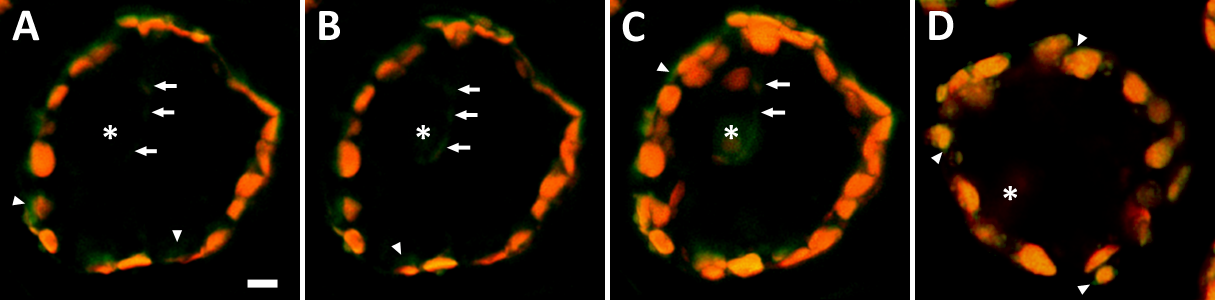

Supplement: Figure S1 — LSCM showing the actin labelling with phalloidin-FITC (A–D). A control cell (latrunculin untreated alga) can be seen in two different focal planes (A,B) and the sum of the focal planes obtained is also shown (C). The figure D corresponds to a latrunculin B treated cell (sum of focal planes obtained). Microfilaments are represented in green and, in red-orange, the auto-fluorescence of CC and chloroplasts. In the latrunculin untreated cell (A–C), it is possible to observe the microfilament labelling in connection structures (arrows), over the CC region (*) and surrounding the chloroplasts (arrowheads), while in a latrunculin treated cell, the connecting structure was not observed, nor was observed an actin labelling in CC region (*). At this treatment, the phalloidin labelling was seen surrounding the chloroplasts (arrowheads), but weaker than the fluorescence observed in untreated cell (control). Bars = 5 µm. (TIF) [file pone.0063929.s001.tif]

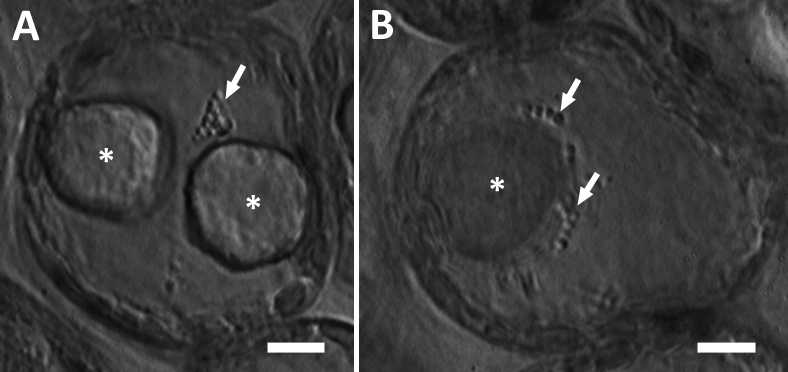

Supplement: Figure S2 — Alga cells double-treated with cytoskeleton depolymerising drugs (both with 1 µM latrunculin and 1.5 mM colchicine) (A,B). * = CC, arrow = vesicles. Note the vesicle accumulation surrounding the CC and the absence of connective structures between the CC and the cell periphery (C). Bar = 3 µm. (TIF) [file pone.0063929.s002.tif]
