# Supplementary material for: Traffic of Secondary Metabolites to Cell Surface in the Red Alga Laurencia dendroidea Depends on a Two-Step Transport by the Cytoskeleton
Source: PLoS One. 2013 May 21;8(5):e63929. doi: 10.1371/journal.pone.0063929 (PMC3660350; doi:10.1371/journal.pone.0063929)
Supplement: Table S1 — Mean values of the number of vesicles accumulated in response to different drug treatments (latrunculin, colchicine and latrucunlin+colchicine). The vesicles accumulation were observed in regions surrounding the CC (in algae cells treated with latrunculin and treated with both drugs simultaneously) and at the cell periphery (in algae cells treated with colchicine). Standard deviations are given after the mean values. The asterisk indicates significant difference between the control and the drug treatments (p<0.01). (DOCX) [file pone.0063929.s003.docx]

**Table S1**: Mean values of the number of vesicles accumulated in response to different drug treatments (latrunculin, colchicine and latrucunlin + colchicine). The vesicles accumulation were observed in regions surrounding the CC (in algae cells treated with latrunculin and treated with both drugs simultaneously) and at the cell periphery (in algae cells treated with colchicine). Standard deviations are given after the mean values. The asterisk indicates significant difference between the control and the drug treatments (p < 0.01).

| Treatment | Accumulated vesicles *per* cell |
| --- | --- |
| Untreated algae (control) | 1.0 ± 1.05 |
| Latrunculin [0.1 mM] | 3.9 ± 2.27 |
| Latrunculin [1 mM] | 11.6 ± 2.64* |
| Colchicine [0.15 mM] | 4.4 ± 2.79 |
| Colchicine [1.5 mM] | 17.5 ± 4.25* |
| Latrunculin [1 mM] + Colchicine [1.5 mM] | 21.4 ± 7.18* |
